# Development and application of a qPCR-based genotyping assay for *Ophidiomyces ophidiicola* to investigate the epidemiology of ophidiomycosis

**DOI:** 10.1371/journal.pone.0289159

**Published:** 2023-08-03

**Authors:** Ellen Haynes, Jeffrey Lorch, Matthew C. Allender

**Affiliations:** 1 Wildlife Epidemiology Laboratory, Department of Veterinary Clinical Medicine, University of Illinois College of Veterinary Medicine, Urbana, Illinois, United States of America; 2 Current affiliation: Southeastern Cooperative Wildlife Disease Study, Department of Population Health, University of Georgia College of Veterinary Medicine, Athens, Georgia, United States of America; 3 U.S. Geological Survey, National Wildlife Health Center, Madison, Wisconsin, United States of America; 4 Chicago Zoological Society, Brookfield Zoo, Brookfield, Illinois, United States of America; City University of New York, UNITED STATES

## Abstract

Ophidiomycosis (snake fungal disease) is an infectious disease caused by the fungus *Ophidiomyces ophidiicola* to which all snake species appear to be susceptible. Significant variation has been observed in clinical presentation, progression of disease, and response to treatment, which may be due to genetic variation in the causative agent. Recent phylogenetic analysis based on whole-genome sequencing identified that *O*. *ophidiicola* strains from the United States formed a clade distinct from European strains, and that multiple clonal lineages of the clade are present in the United States. The purpose of this study was to design a qPCR-based genotyping assay for *O*. *ophidiicola*, then apply that assay to swab-extracted DNA samples to investigate whether the multiple *O*. *ophidiicola* clades and clonal lineages in the United States have specific geographic, taxonomic, or temporal predilections. To this end, six full genome sequences of *O*. *ophidiicola* representing different clades and clonal lineages were aligned to identify genomic areas shared between subsets of the isolates. Eleven hydrolysis-based Taqman primer-probe sets were designed to amplify selected gene segments and produce unique amplification patterns for each isolate, each with a limit of detection of 10 or fewer copies of the target sequence and an amplification efficiency of 90–110%. The qPCR-based approach was validated using samples from strains known to belong to specific clades and applied to swab-extracted *O*. *ophidiicola* DNA samples from multiple snake species, states, and years. When compared to full-genome sequencing, the qPCR-based genotyping assay assigned 75% of samples to the same major clade (Cohen’s kappa = 0.360, 95% Confidence Interval = 0.154–0.567) with 67–77% sensitivity and 88–100% specificity, depending on clade/clonal lineage. Swab-extracted *O*. *ophidiicola* DNA samples from across the United States were assigned to six different clonal lineages, including four of the six established lineages and two newly defined groups, which likely represent recombinant strains of *O*. *ophidiicola*. Using multinomial logistic regression modeling to predict clade based on snake taxonomic group, state of origin, and year of collection, state was the most significant predictor of clonal lineage. Furthermore, clonal lineage was not associated with disease severity in the most intensely sampled species, the Lake Erie watersnake (*Nerodia sipedon insularum*). Overall, this assay represents a rapid, cost-effective genotyping method for *O*. *ophidiicola* that can be used to better understand the epidemiology of ophidiomycosis.

## Introduction

Ophidiomycosis (snake fungal disease) is a threat to the health of free-ranging snakes and those under human care worldwide [1–3]. All species of snakes appear to be susceptible [4] and the disease commonly manifests as skin lesions [2, 3, 5], but internal visceral disease also has been documented [6–10]. The causative agent, *Ophidiomyces ophidiicola*, has been established via experimental infection studies in snakes from the families Viperidae and Colubridae [5, 11, 12]. This keratinophilic fungus utilizes a variety of carbon and nitrogen sources and can survive and grow under a wide range of environmental conditions [13]. Recent work isolated *O*. *ophidiicola* from the soil of snake hibernacula, but found that fungal growth was inhibited by microbial communities naturally found in soil, indicating that this is a specialized pathogen of snakes that uses soil as an environmental reservoir [14].

Molecular diagnostics and genetic analyses are increasingly important tools for understanding the epidemiology of emerging infectious diseases, including the success of treatment and management approaches [15]. Previous phylogenetic analyses placed *O*. *ophidiicola* isolates from wild European snakes in a different clade than isolates from wild North American snakes, whereas *O*. *ophidiicola* isolates from snakes under human care and a wild snake from Taiwan were assigned to a third clade [16, 17]. Furthermore, differences in the fungal growth rate in culture were found between the strains isolated from wild European and North American snakes and between strains isolated from a plains garter snake (*Thamnophis radix*) and an eastern massasauga rattlesnakes (*Sistrurus catenatus*), both from the state of Illinois [13, 16]. Within the United States, differences in pathogen genetics and disease characteristics indicate that multiple lineages are present that could vary in virulence [13, 17]. Additionally, the current literature has identified significant variation in the presentation of ophidiomycosis across species and between individual snakes [6, 7, 9, 18–21]. In clinical trials, differences in response to treatment between snakes have been observed [13, 22], but it is unclear if these are due to genomic variation among *O*. *ophidiicola* strains. Therefore, it is crucial to determine how different fungal strains may be impacting snakes across the United States.

Numerous DNA fingerprinting techniques have been used to identify different strains of other fungal pathogens. These include restriction fragment length polymorphisms (RFLP), RFLP with hybridization probes, PCR-based methods, electrophoretic karyotyping, and sequencing-based methods [23]. Molecular methods for analyzing fungal pathogens, including genotyping, have been crucial for tracing disease outbreaks in humans [24, 25] as well as in wildlife species, specifically with *Batrachochytrium dendrobatidis* (*Bd*), the causative fungus of amphibian chytridiomycosis [26, 27]. A multiplex qPCR-based genotyping assay specific for *Bd* was developed and optimized for the low quantity of DNA often obtained from skin swabbing [28]. This study found that the analysis of swab-extracted DNA replicated the findings from whole-genome data in its ability to differentiate among the major *Bd* clades. Application of this assay has enabled non-invasively collected skin swabs to be used to characterize the epidemiology of *Bd* and its effects on declining amphibian populations in a large-scale, cost-effective manner. Multiplex qPCR assays have also been designed to differentiate between strains of human parvovirus B19 [29], hepatitis viruses [30], JC virus [31], porcine circovirus-2 [32], human papillomavirus [33], *Giardia lamblia* [34], and *Theileria equi* [35].

The purpose of this study was to design a qPCR-based genotyping assay for distinguishing major clades and clonal lineages of *O*. *ophidiicola*, then apply that assay to swab-extracted DNA samples to investigate whether geographic, host taxonomic, or temporal patterns are associated with these lineages in the United States. We predicted that our assay would have high sensitivity and specificity for clade assignment, compared to whole genome sequencing. We also predicted that different clades of *O*. *ophidiicola* are associated with specific snake species, geographic areas, and sampling years. Overall, understanding the genomic variation and epidemiology of *O*. *ophidiicola* across the United States will enable veterinarians and wildlife managers to more effectively treat individual snakes and design management plans to control the spread of ophidiomycosis in wild snakes and those under human care.

## Materials and methods

### Genomic analysis

The assemblies of six complete genome sequences of *O*. *ophidiicola* isolates representing six distinct clades and clonal lineages of the fungus (clades I and III and four clonal lineages of clade II: IIA, IID, IIE, and IIF) were obtained from the U.S. Geological Survey National Wildlife Health Center (GenBank accession numbers: CM040670.1, CM040681.1, CM040684.1, CM040675.1, CM040695.1, CM040663.1). These clades and clonal lineages were determined based on a phylogenetic analysis of 82 *O*. *ophidiicola* isolates [17]. Whole-genome sequences were aligned using the progressiveMauve alignment algorithm in Mauve Multiple Genome Alignment software version 2.4.0 [36], then the backbone color scheme display mode was used to identify 29 genomic areas shared between subsets of the isolates with 100% sequence similarity. Nucleotide sequences from these genomic areas were obtained using Geneious® version 11.1.4 [37].

### Primer design

TaqMan primer-probe sets were designed using Primer Express software (ThermoFisher Scientific, Waltham, Massachusetts, USA) to target 50–100 base pair regions within the previously identified genomic areas (S1 Table). Primer-probe sets with optimal conditions for the Fluidigm Access Array System (Fluidigm, South San Francisco, California, USA) were tested *in silico* for specificity for *O*. *ophidiicola* and the target isolates. The Basic Local Alignment Tool (BLAST) in Geneious® was used to search for the sequences targeted by each primer-probe set within the six *O*. *ophidiicola* isolate genomes. The NCBI BLASTN query was used to search for target sequence matches in other organisms and the NCBI Primer-BLAST tool was used to search for matches to the forward and reverse primers. The 14 primer-probe sets that had the highest specificity and that targeted both conserved and divergent sequences among the six isolates, such that each isolate had its own unique set of targeted areas (Table 1), were selected for additional testing. Each primer-probe set was tested for its limit of detection for the DNA sequence of interest through qPCR with a dilution series from 10^7^ target copies to 10^0^ copies of custom DNA fragments containing the target sequences (Invitrogen GeneArt High-Q Strings, ThermoFisher Scientific, Waltham, Massachusetts, USA). Reactions were run in triplicate and a dilution was considered positive if at least one replicate amplified. Linear, double-stranded DNA fragments were reconstituted from dried pellets using nuclease-free water, according to the manufacturer’s instructions, to a concentration of 10^10^ copies. Number of copies in the initial pellet was calculated based on each fragment’s length and the pellet mass, assuming an average molar mass of 650 Daltons per base pair, using an online calculator through the University of Rhode Island Genomics and Sequencing Center (http://cels.uri.edu/gsc/cndna.html). The 11 primer-probe sets with the lowest limits of detection were selected for the genotyping assay. Primer and probe sequences are provided in Table 2.

**Table 1:** Target sequence amplification patterns for each of six *Ophidiomyces ophidiicola* clades/clonal lineages.

			Primer-probe Targets								
Clade/Clonal Lineage	A	B	C	D	E	F	G	H	I	J	K
Clade I SAMN23192525		x	x	x				x			
Clonal Lineage IIA SAMN23192514	x	x		x^a^	x	x	x	x	x		
Clonal Lineage IID SAMN23192510	x	x	x	x				x	x		x
Clonal Lineage IIE SAMN23192520	x	x		x				x		x	
Clonal Lineage IIF SAMN23192500		x		x				x	x	x	x
Clade III SAMN23192534			x	x	x	x	x				
Recombinant Group 1	x	x	x	x				x	x	x	x
Recombinant Group 2	x	x	x	x	x	x	x	x	x		x

Clades/clonal lineages are based on phylogenetic analysis (clades I, II, and III and clonal lineages IIA, IID, IIE, and IIF), with two additional groups based on unique sample amplification patterns identified in the present study (Recombinant Group 1 and 2). GenBank biosample numbers are provided for the representative sequence that was used for each clade/clonal lineage during assay design. The letters correspond to each of the 11 primer-probe targets used for the genotyping assay.

^a^Target not found in the genome assembly, but present in the Sequence Read Archive (SRA) data.

**Table 2:** Primer and probe sequences for each of the 11 genotyping targets.

Target ID	Forward primer	Reverse primer	Probe
A	AGCCCCATTGCGCAAGTA	CCCGTTGCCATTTATAGAAGCA	CGAGACAAGAGATACTC
B	CGCGCCGGCTGTGT	AGGGAGCTGGCCCTCTTTATA	CGGTTATAGTTCACCAGCC
C	TCATTTGCTCGGTCCTGCTT	TCCCGAGCTTTTGGAACTGT	ACTGCTAGTTGCACCCTA
D	AGCGTTTTGCCCCAGCTA	GCAATGTTGCTCCGCAGTACT	CTCGACTACGAAATCG
E	TCAGGCGATGGGAAATGGT	AATGGCATGCGCGCTATC	TGCCCCAAAGATG
F	TCACGGCTGCAGTCTCATAAA	TCGTGGAGCTGATCGGTAGAG	AGAGATCCTCATCCTTG
G	CCATCCTGCGCAACTACAAAA	GAGTGGCTGTCAGTGTTTGCA	ACCTTGTTGTTAGCCATGG
H	AGTGTTGACACCCCCCCTACT	TGAGCAACTTGGTTGTGAAAAGA	TTACACCAAAACCC
I	CGAAACGAACGCCCAACT	CATCATACCGCCTATCCGAAA	TTCCTGGTCCTGTGCGG
J	CAACGCACTGCCGTTTAGG	AGCGGAAGGCTGAGATTCAG	CTCTTCCCGCGACCTA
K	AGATGCTCGTCCCTCTTCGA	GGAAGCGTGGTAGCCTGATG	CCCTTCTCCATGTTCCT

### *Ophidiomyces ophidiicola* genotyping assay design

The TaqMan primers described above were used in a pre-amplification protocol to enrich the DNA template, which improves the success of downstream PCR when working with the low quantity of DNA typical of skin swab samples [28]. In the pre-amplification protocol, sample DNA was combined with the pooled primers and TaqMan PreAmp Master Mix (Applied Biosystems, Life Technologies, Austin, Texas, USA) then subjected to amplification according to the manufacturer’s instructions. Following pre-amplification, multiple simplex qPCR reactions were performed simultaneously using the Fluidigm Access Array platform with the primer-probe sets described above. The primer-probe set for *O*. *ophidiicola* that was originally used to identify positive samples [38] was included to confirm the previous testing. Primer-probes for *Aspergillus* spp. [39], *Batrachochytrium dendrobatidis* [40], *Batrachochytrium salamandrivorans* [41], *Emydomyces testavorans* [42], *Fusarium* spp. [43],and *Pseudogymnoascus destructans* [44], as well as corresponding positive controls, were included to test the specificity of the genotyping assay and to detect additional fungal species in the DNA extracts from snake skin swabs. Samples were considered positive if they had a Fluidigm mean cycle threshold (C_t_) value less than 24 and DNA samples were assigned to *O*. *ophidiicola* clades based on their amplification patterns (Table 1). Samples with non-specific amplification patterns were examined for targets with C_t_ values between 24 and 27 and single target qPCR was performed to assist with clade assignment.

### *Ophidiomyces ophidiicola* genotyping assay validation

The qPCR-based genotyping assay was assessed by comparing clade assignments based on that assay to clade assignments based on whole-genome sequence data of the same *O*. *ophidiicola* isolates [17]. A total of 82 DNA extracts were used, including samples from each of the six representative clades, putative recombinant strains, and fungi other than *O*. *ophidiicola* (Table 3). The qPCR results were interpreted by a single blinded observer, then compared to clade assignments based on whole-genome sequencing results. Cohen’s kappa coefficient [45] was calculated to assess agreement between the two methods; sensitivity and specificity were calculated for the qPCR assay for each clade, as well as the clonal lineages of clade II, using whole-genome sequencing as the reference method.

**Table 3:** Clade or clonal lineage assignment based on qPCR and whole-genome sequencing [17] for 82 DNA extracts of *Ophidiomyces ophidiicola* and other fungi.

Sample ID	qPCR assigned clade or clonal lineage	Whole-genome sequencing assigned clade or clonal lineage	Clade or clonal lineage based on BLAST-based genotyping	Sample details
45707–81	I	I		
45707–82	I	I		
45707–83	I	I		
45692–2	Recombinant Group 2	I	I*	
UAMH-6688	III	III		
IAMH-10296	III	III		
UAMH-9985	IID	III	Recombinant Group 2*	
44736–89	Recombinant Group 2	IIA	IIA*	
CBS 122913	Initially Recombinant Group 2; Reassigned to IIA	IIA	IIA	
24392–1	IID	IID		
24395–1	IID	IID		
24414–1	IID	IID		
24415–1	IID	IID		
24746–1	IID	IID		
24824–1	IID	IID		
24828–1	IID	IID		
24900–1	IID	IID		
26480–1	IID	IID		
26583–2	IID	IID		
27239–1	IID	IID		
27242–2	IID	IID		
27242–3	IID	IID		
44781–8	IID	IID		
44736–52	IID	IID		
44736–65	IID	IID		
44736–88	IID	IID		
UAMH-6218	IID	IID		
24564–1	IID	IID		
27422–1	IID	IID		
24874–1	Recombinant Group 1	IID	IID*	
27242–4	Recombinant Group 1	IID	IID*	
24852–1	Recombinant Group 1	IID	IID*	
24885–1	Recombinant Group 1	IID	IID*	
22687–1	IIE	IIE		
44736–31	IIE	IIE		
44781–25	IIE	IIE		
22747–6	IIE	IIE		
26452–1	IIE	IIE		
44736–14	IIE	IIE		
23906–1	Recombinant Group 1	IIE	IIE*	
24281–1	IIF	IIF		
UAMH-10949	IIF	IIF		
UAMH-11295	IIF	IIF		
27466–1	Recombinant Group 1	IIF	IIF*	
44736–95	IID	IID, IIE	IID	Recombinant
26341–1	IID	IID, IIE	IID	Recombinant
44736–86	IID	IID, IIE	IID	Recombinant
44736–90	IID	IID, IIE	IID	Recombinant
UAMH-6642	IID	IID, IIE	IID	Recombinant
44781–26	IID	IID, IIE	IID	Recombinant
24393–1	Recombinant Group 1	IID, IIE	Recombinant Group 1	Recombinant
44736–94	Recombinant Group 1	IID, IIE	Recombinant Group 1	Recombinant
23942–1	IIF	IID, IIF (IIB)	IIF	Recombinant
24878–1	IIF	IID, IIF (IIC)	IIF	Recombinant
26465–1	IIF	IID, IIF (IIB)	IIF	Recombinant
23913–1	IIF	IID, IIF (IIB)	IIF	Recombinant
24878–5	IIF	IID, IIF (IIC)	IIF	Recombinant
24266–6	IIF	IID, IIF	IIF	Recombinant
24042–1	IID	IID, IIF	IID	Recombinant
24266–1	IID	IID, IIF	IID	Recombinant
24266–5	IID	IID, IIF	IID	Recombinant
24411–1	IID	IID, IIF	IID	Recombinant
26671–1	IID	IID, IIF	IID	Recombinant
44781–7	IID	IID, IIF	IID	Recombinant
24894–1	IID or IIF	IID, IIF	IID or IIF	Recombinant
24825–2	Recombinant Group 1	IID, IIF (IIB)	IIF*	Recombinant
24878–6	Recombinant Group 1	IID, IIF (IIC)	IIF*	Recombinant
27421–1	Recombinant Group 1	IID, IIF	Recombinant Group 1*	Recombinant
44736–45	Recombinant Group 1	IID, IIF	IIF*	Recombinant
UAMH-10768	Recombinant Group 1	IID, IIF	Recombinant Group 1	Recombinant
CBS 102663	Recombinant Group 1	IID, IIF	Recombinant Group 1	Recombinant
44781–4	Recombinant Group 1	IID, IIF	IIF*	Recombinant
24266–2	IIF	IID, IIE, IIF	IID or IIF	Recombinant
44736–93	IID	IID, IIE, IIF	IID	Recombinant
44736–87	IID	IID, IIE, IIF	IID	Recombinant
24266–3	IID or IIF	IID, IIE, IIF	IID or IIF	Recombinant
44736–75	Recombinant Group 2	IIA, IID, unsampled lineage	Recombinant Group 2*	Recombinant
UAMH-3527	No target amplification	non-*Ophidiomyces*		*Nannizziopsis vriesii*
UAMH-10212	No target amplification	non-*Ophidiomyces*		*Chrysosporium indicum*
UAMH-10352	No target amplification	non-*Ophidiomyces*		*Nannizziopsis guarroi*
UAMH-11645	No target amplification	non-*Ophidiomyces*		*Paranannizziopsis australasiensis*
UAMH-3880	No target amplification	non-*Ophidiomyces*		*Uncinocarpus reesii*

Results of BLAST-based genotyping (based on searching for the target sequences in the assembled genomes of each sample in the National Center for Biotechnology Information database) are provided for samples where qPCR and whole-genome sequencing clade assignments disagreed. Asterisks indicate when the BLAST-based pattern differed from the pattern produced by qPCR.

Following publication of the whole-genome sequences of the 82 DNA extracts used for assay validation, BLAST-based genotyping was performed for samples that had disagreement between the qPCR-based and whole-genome sequencing-based approaches, as well as samples that were classified as recombinant (n = 43, Table 3). This approach involved searching for each of the 11 target sequences (S2 Table) within the genome assemblies available on the National Center for Biotechnology Information (NCBI) database using the BLAST assembly function. The resulting pattern for each sample was then used to assign it to a clade or clonal lineage based on the same criteria described above and was compared to the amplification pattern produced by the qPCR-based assay to investigate whether the targets responsible for incorrect classification were present or absent in the genome of that sample.

### *Ophidiomyces ophidiicola* genotyping assay application

A total of 607 snake skin swab samples positive for *O*. *ophidiicola* DNA were selected for genotyping. All samples previously tested positive for *O*. *ophidiicola* using a specific qPCR protocol targeting the internal transcribed spacer (ITS) region of ribosomal DNA [38]. The selected samples represented 31 snake species from 13 states and the District of Columbia. Samples were collected between 2000 and 2020 and stored at -80°C for up to 20 years before genotyping. DNA extraction from swabs of snake skin followed the manufacturer’s recommendations (QIAamp DNA Mini Kit, Qiagen Inc., Valencia, California, USA) with the addition of a 1-hour incubation at 37°C with 12.5U of lyticase (Sigma-Aldrich, St. Louis, Missouri, USA), prior to the lysis step, to break down the fungal cell wall. Following DNA extraction, each sample was assessed for DNA quantity (measured in ng/°l) and quality (using the ratio of absorbance at 260 nm to 280 nm) using spectrophotometry (Nanodrop1000, ThermoFisher Scientific, Wilmington, Delaware, USA).

### Statistical analysis

Logistic regression models were used to predict amplification sufficient for clade/clonal lineage assignment and to produce receiver operating characteristic (ROC) curves based on DNA concentration, *O*. *ophidiicola* ITS copies per reaction as determined using qPCR, and years of sample storage (JMP® Pro, Version 14.2.0, SAS Institute Inc., Cary, North Carolina, USA). *Ophidiomyces ophidiicola* copies per reaction values were based on the initial testing of each sample using the specific qPCR assay [38].

For each *O*. *ophidiicola* DNA sample, the host species, state, subpopulation, and year of collection were compiled. A series of univariable and multivariable multinomial logistic regression models were built with *O*. *ophidiicola* clade as the categorical output variable and host snake family, subfamily, genus, and species, as well as year and state as the predictor variables (function multinom, R package nnet [46]). Variables with significant pairwise comparisons in univariable models were included in multivariable models and model ranking was performed using an information-theoretic approach based on the Akaike information criterion (AIC). Factor levels with fewer than five samples were excluded and a Bonferroni correction was used for multiple statistical comparisons. Risk ratios and 95% confidence intervals were calculated for significant comparisons. This analysis was conducted in RStudio Version 1.2.1335 [47], with statistical significance assessed at α = 0.05.

Data from Lake Erie watersnakes were analyzed for associations between clinical disease severity and *O*. *ophidiicola* clade/clonal lineage; this was the species for which disease severity data were consistently available. Disease severity metrics (presence/absence of lesions, number of lesions, most severe lesion type, and severity category) were determined for individual snakes at the time of swab collection. Severity category (mild, moderate, severe) was based on disease severity scores, as determined using a previously published scoring system incorporating lesion type, size, location, and number [2]. Animals with scores of six or less were categorized as mildly affected, scores between seven and nine were classified as moderate, and scores of 10 or greater were classified as severe. The association between number of lesions and clade/clonal lineage was evaluated using a Kruskal-Wallis test, whereas associations between lesion presence/absence, lesion type, and severity category were evaluated using Fisher’s Exact test (JMP® Pro, Version 14.2.0, SAS Institute Inc., Cary, North Carolina, USA), with statistical significance assessed at α = 0.05.

## Results

### *Ophidiomyces ophidiicola* genotyping assay design and validation

Eleven primer-probe sets were selected for the genotyping assay that produced unique amplification patterns for each of the three major clades (clades I, II, and III) and four clonal lineages within clade II (IIA, IID, IIE, and IIF; Table 1) [17]. Specifically, each clade or clonal lineage had a different set of primer-probe target sequences present in its genome. The selected primer-probe sets were able to detect 10 or fewer copies of the target sequence and had a qPCR reaction efficiency of 90–110% (S1 Table). No matches to other organisms were found for either the target sequences or the primer sets. For the 77 strains of *O*. *ophidiicola* previously assigned to a clade or clonal lineage based on whole-genome sequencing, the qPCR-based assay assigned 75% of the samples to the correct major clade (Cohen’s kappa = 0.360, 95% confidence interval (CI) = 0.154–0.567) (Table 3). Using whole-genome sequencing as the reference, the qPCR assay was 75% (95% CI = 19–99%) sensitive and 100% (95% CI = 95–100%) specific for clade I, 77% (95% CI = 65–86%) sensitive and 88% (95% CI = 47–100%) specific for clade II, and 67% (95% CI = 9–99%) sensitive and 100% (95% CI = 95–100%) specific for clade III. For the 37 samples assigned to single clonal lineages of clade II based on whole-genome sequencing (i.e., non-recombinant samples), there was 81% agreement between the two methods in clonal lineage assignment. For the clonal lineages within clade II, including only the non-recombinant samples (n = 37), the qPCR-based assay was 50% (95% CI = 1–99%) sensitive and 100% (95% CI = 92–100%) specific for clonal lineage IIA, 83% (95% CI = 63–95%) sensitive and 95% (95% CI = 75–100%) specific for clonal lineage IID, 86% (95% CI = 42–100%) sensitive and 100% (95% CI = 91–100%) specific for clonal lineage IIE, and 75% (95% CI = 20–99%) sensitive and 100% (95% CI = 91–100%) specific for clonal lineage IIF. All but one of the cases of non-agreement involved additional target amplification, resulting in assignment to one of the newly defined recombinant groups (Table 3). A single sample from clade III was assigned to clonal lineage IID based on lack of target amplification. In cases where whole-genome sequencing identified isolates as recombinant samples between two or more clonal lineages within clade II, the qPCR assay generated amplification profiles of one of the known parental lineages or produced a novel “recombinant group” profile that included amplification of targets present in both parent lineages.

BLAST-based genotyping results (Table 3) indicated that in eight of the 10 cases of non-agreement among non-recombinant samples, the additional targets that amplified with qPCR were not present in the sample genome (i.e., false positive on qPCR). Furthermore, in these cases, the amplification pattern based on BLAST resulted in the same clade assignment as whole-genome sequencing. For one of the other two samples with non-agreement (CBS 122913), the additional target detected with qPCR (Target D) was found in the genome sequence, resulting in disagreement with the whole genome sequencing-based assignment. However, further interrogation of the Sequence Read Archive (SRA) files in GenBank (SRR17214499) for the strain of clade IIA (44736–89) used to design the assay showed that target D was indeed present in that strain, which changed the defined amplification pattern for clade IIA (Table 1). Therefore, although sample CBS 122913 was initially assigned to recombinant group 2, it was reassigned to clade IIA based on the updated pattern of that clade. For the last sample (UAMH-9985), one target was detected with qPCR, but not found in the genome, whereas two targets were detected with the genome but not by qPCR, resulting in disagreement across all three approaches. The most common false positive targets on qPCR were target K (n = 4 samples), target J (n = 4 samples), and target C (n = 2 samples). Targets A and E were each false positive on qPCR for a single sample. The only targets that were present in the NCBI genomes but missed by qPCR were target E (n = 1) and target H (n = 1); both were missed in the same sample. For the recombinant samples (n = 33), the amplification pattern based on the BLAST approach matched the qPCR-based pattern for all but six samples. For those samples, targets A (n = 1), B (n = 1), and C (n = 4) were qPCR false positives, whereas target K (n = 1) was found in the genome but did not amplify with qPCR.

### *Ophidiomyces ophidiicola* genotyping assay performance

**Fig 1:**
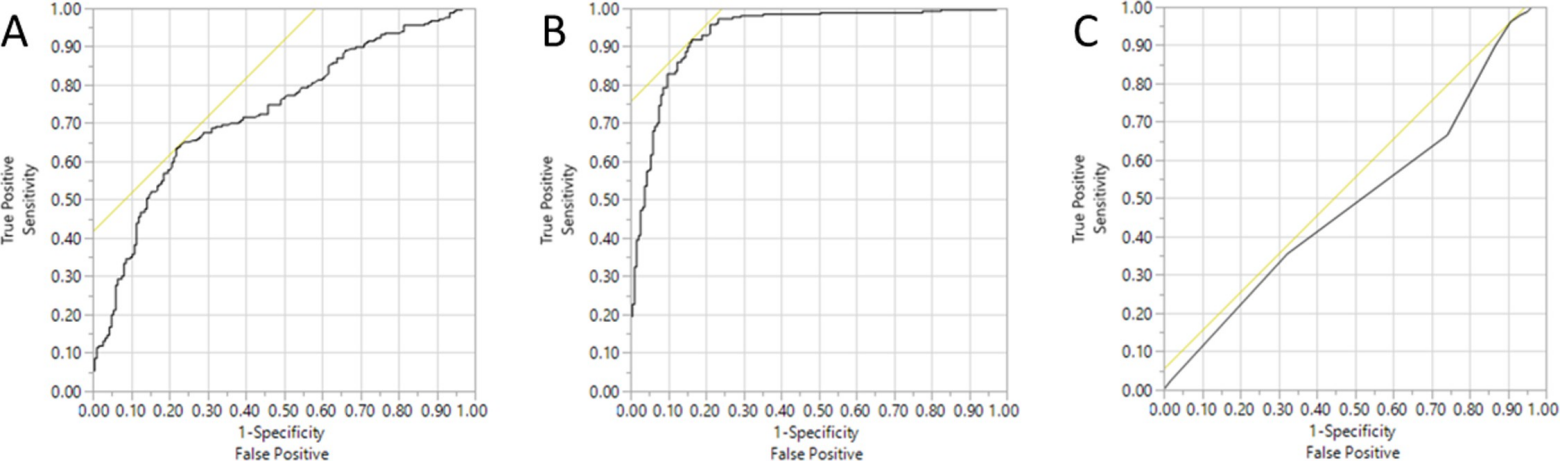
Receiver operating characteristic (ROC) curves for predicting sufficient qPCR amplification for *Ophidiomyces ophidiicola* clade assignment. Curves are based on (A) DNA concentration (AUC = 0.73), (B) copies of ITS of *Ophidiomyces ophidiicola* per qPCR reaction (AUC = 0.94), and (C) years of sample storage (AUC = 0.49).

Out of the 607 skin swab samples that were previously positive for *O*. *ophidiicola* based on ITS qPCR and submitted for genotyping, 336 samples (55%) had sufficient amplification of the genotyping targets for clade assignment, 30 samples had failed amplification of the primers for the ITS region of *O*. *ophidiicola* and all the genotyping primers, and 241 samples had amplification of the *O*. *ophidiicola* ITS region but lacked amplification of genotyping primers sufficient for clade assignment. Specifically, 124 samples had no genotyping targets amplify, whereas 117 had non-specific target amplification. DNA concentration was a significant predictor for amplification sufficient for clade assignment (p<0.0001), and a cutoff value of 6.3 ng/°L was 63% sensitive and 78% specific for differentiating between specific and non-specific amplification (Area Under the Curve (AUC) = 0.73), indicating that samples with low DNA concentrations are less likely to be assigned to a clade (Fig 1A). Quantity of *O*. *ophidiicola* per qPCR reaction was also predictive of clade assignment success (p<0.0001), and a cutoff value of 187.4 ITS copies per reaction was 92% sensitive and 84% specific for predicting successful clade assignment (AUC = 0.93), indicating that samples with more *O*. *ophidiicola* DNA were more likely to be assigned to a clade (Fig 1B). Length of storage was positively associated with likelihood of clade assignment (p = 0.0092), indicating that older samples were more likely to be assigned to a clade; the cutoff value of 4 years was 96% sensitive but only 9% specific (AUC = 0.49), indicating poor predictive value for this factor (Fig 1C).

**Fig 2:**
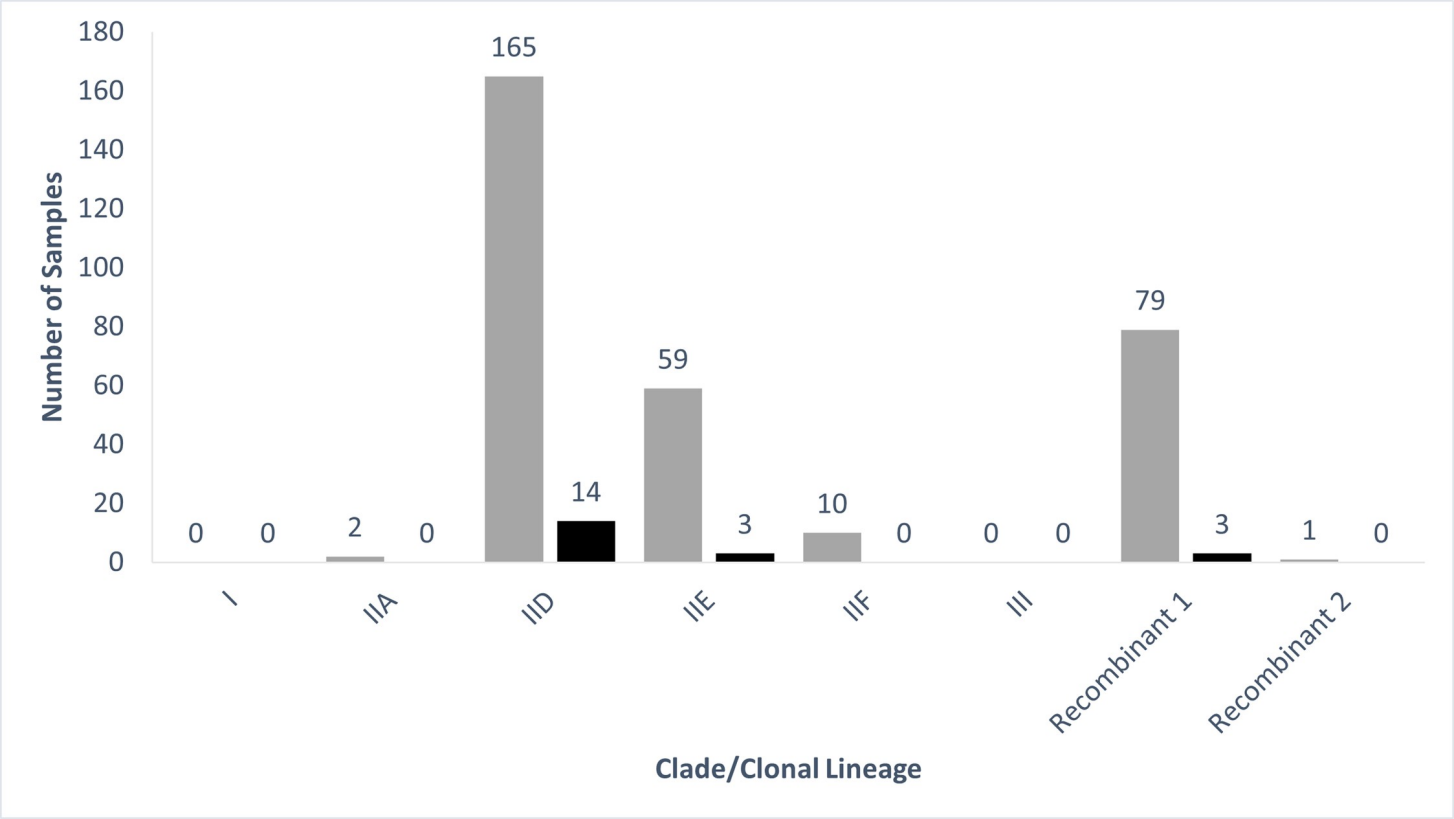
Bar graph of number of snake skin swab-extracted DNA samples assigned to each of eight *Ophidiomyces ophidiicola* clades or clonal lineages. All samples previously tested qPCR positive for *O*. *ophidiicola* and were collected from wild snakes (gray bars on the left of each pair of bars) or snakes from managed collections (black bars on the right of each pair of bars).

Two new amplification patterns were defined based on the unique patterns of primer-probe target amplification for certain swab samples and are herein described as recombinant groups 1 and 2 (Table 1). Specifically, the amplification pattern for recombinant group 1 resembled that of clonal lineage IID with the addition of target J, which is present in clonal lineages IIE and IIF; this indicates putative recombination between lineage IID and IIE or IIF. The amplification pattern for recombinant group 2 resembled the amplification pattern of clonal lineage IIA with the addition of amplification targets C and K (Table 1). Recombinant group 2 also produced a pattern identical to the previously described strain NWHC 44736–75, which is believed to be of hybrid origin [17]. For both snakes in managed collections (n = 20) and wild snakes (n = 316), most samples were assigned to clonal lineage IID, followed by recombinant group 1, then clonal lineage IIE, then clonal lineage IIF, then clonal lineage IIA, and finally recombinant group 2 (Fig 2). No samples were assigned to clade I, or clade III. Clade was assigned to samples collected from 27 species (Table 4), 11 states (Table 5), and eight years (Table 6).

**Table 4:** Number of snake skin swab-extracted DNA samples that tested qPCR positive for *Ophidiomyces ophidiicola* from 27 snake species successfully genotyped and assigned to each clade/clonal lineage using the qPCR-based assay.

Species	Total	Clonal Lineage IIA	Clonal Lineage IID	Clonal Lineage IIE	Clonal Lineage IIF	Recombinant Group 1	Recombinant Group 2
*Agkistrodon piscivorus*	9	--	7	--	--	1	1
*Carphophis amoenus amoenus*	13	--	10	--	1	2	--
Clonophis kirtlandii*	2	--	--	2	--	--	--
*Coluber constrictor*	16	--	12	4	--	--	--
*Crotalus adamanteus*	15	--	4	5	3	3	--
*Crotalus horridus*	31	--	13	3	3	12	--
Diadophis punctatus*	6	--	6	--	--	--	--
*Drymarchon couperi*	9	--	4	--	--	5	--
Lampropeltis calligaster*	5	--	--	--	--	5	--
Lampropeltis getula*	1	--	1	--	--	--	--
*Lampropeltis triangulum*	9	--	5	4	--	--	--
*Nerodia erythrogaster*	7	1	5	1	--	--	--
*Nerodia fasciata*	3	--	2	--	--	1	--
*Nerodia sipedon*	32	--	20	7	2	3	--
*Nerodia sipedon insularum*	86	--	46	19	--	23	--
*Nerodia taxispilota*	8	--	3	--	1	4	--
Opheodrys vernalis*	1	--	1	--	--	--	--
*Pantherophis alleghaniensis*	13	1	5	5	--	2	--
Pantherophis spiloides*	1	--	--	1	--	--	--
Pantherophis vulpinus*	1	--	1	--	--	--	--
*Regina septemvittata*	6	--	3	3	--	--	--
*Sistrurus catenatus*	51	--	25	6	--	20	--
Thamnophis sauritus*	2	--	--	2	--	--	--
*Thamnophis sirtalis*	7	--	6	--	--	1	--

Asterisks indicate that all samples from a species were assigned to a single clade/clonal lineage. Dashes indicate that no samples from a particular species were assigned that group.

**Table 5:** Number of snake skin swab-extracted DNA samples that tested qPCR positive for *Ophidiomyces ophidiicola* successfully genotyped and assigned to each clade/clonal lineage using the qPCR-based assay from wild snakes from 11 different US states and snakes from managed collections.

US State	Total	Clonal Lineage IIA	Clonal Lineage IID	Clonal Lineage IIE	Clonal Lineage IIF	Recombinant Group 1	Recombinant Group 2
**Georgia**	44	--	27	1	1	14	1
**Illinois**	53	--	22	6	--	25	--
**Indiana**	22	--	10	12	--	--	--
Massachusetts*	11	--	11	--	--	--	--
**Maryland**	12	--	4	--	3	5	--
North Carolina*	2	2	--	--	--	--	--
**New York**	4	--	3	1	--	--	--
**Ohio**	136	--	64	34	3	35	--
**South Carolina**	8	--	--	5	3	--	--
Tennessee*	23	--	23	--	--	--	--
Wisconsin*	1	--	1	--	--	--	--
**Managed collections**	20	--	14	3	--	3	--

Asterisks indicate that all samples from a state were assigned to a single clade. Dashes indicate that no samples from a particular state were assigned to that group.

**Table 6:** Number of snake skin swab-extracted DNA samples that tested qPCR positive for *Ophidiomyces ophidiicola* collected in eight different years successfully genotyped and assigned to each clade/clonal lineage using the qPCR-based assay.

Year	Total	Clonal Lineage IIA	Clonal Lineage IID	Clonal Lineage IIE	Clonal Lineage IIF	Recombinant Group 1	Recombinant Group 2
**2013**	4	--	1	1	--	2	--
**2014**	3	--	2	--	--	1	--
**2015**	6	--	--	2	--	4	--
**2016**	21	--	13	4	--	4	--
**2017**	78	--	33	18	1	25	1
**2018**	105	2	60	14	7	22	--
**2019**	111	--	67	23	1	20	--
**2020**	8	--	3	--	1	4	--

Dashes indicate that no samples from a particular state were assigned to that group.

### Clade/clonal lineage distribution

**Fig 3:**
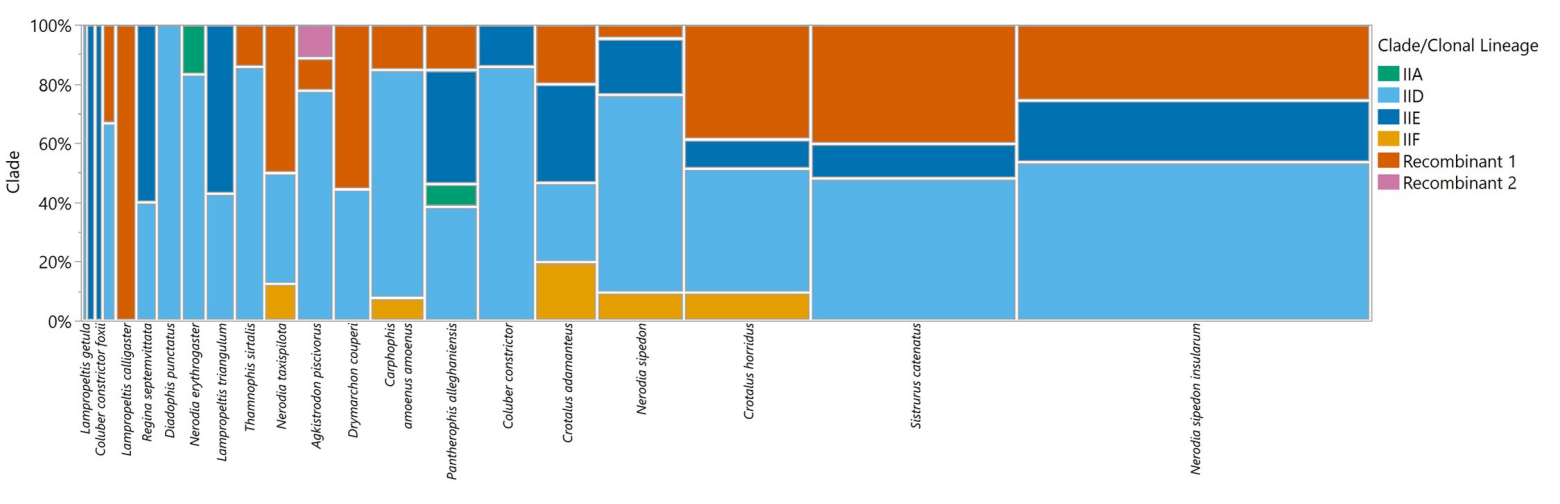
Distribution of *Ophidiomyces ophidiicola* clade/clonal lineage by snake species. Each vertical bar represents a snake species, with the width of the bar proportional to the sample size of that group and species arranged from left to right in order of increasing sample size. A full list of species names with corresponding sample sizes can be found in Table 4. The vertical axis shows the proportion of each clade, noted in a different color, in each group.

**Fig 4:**
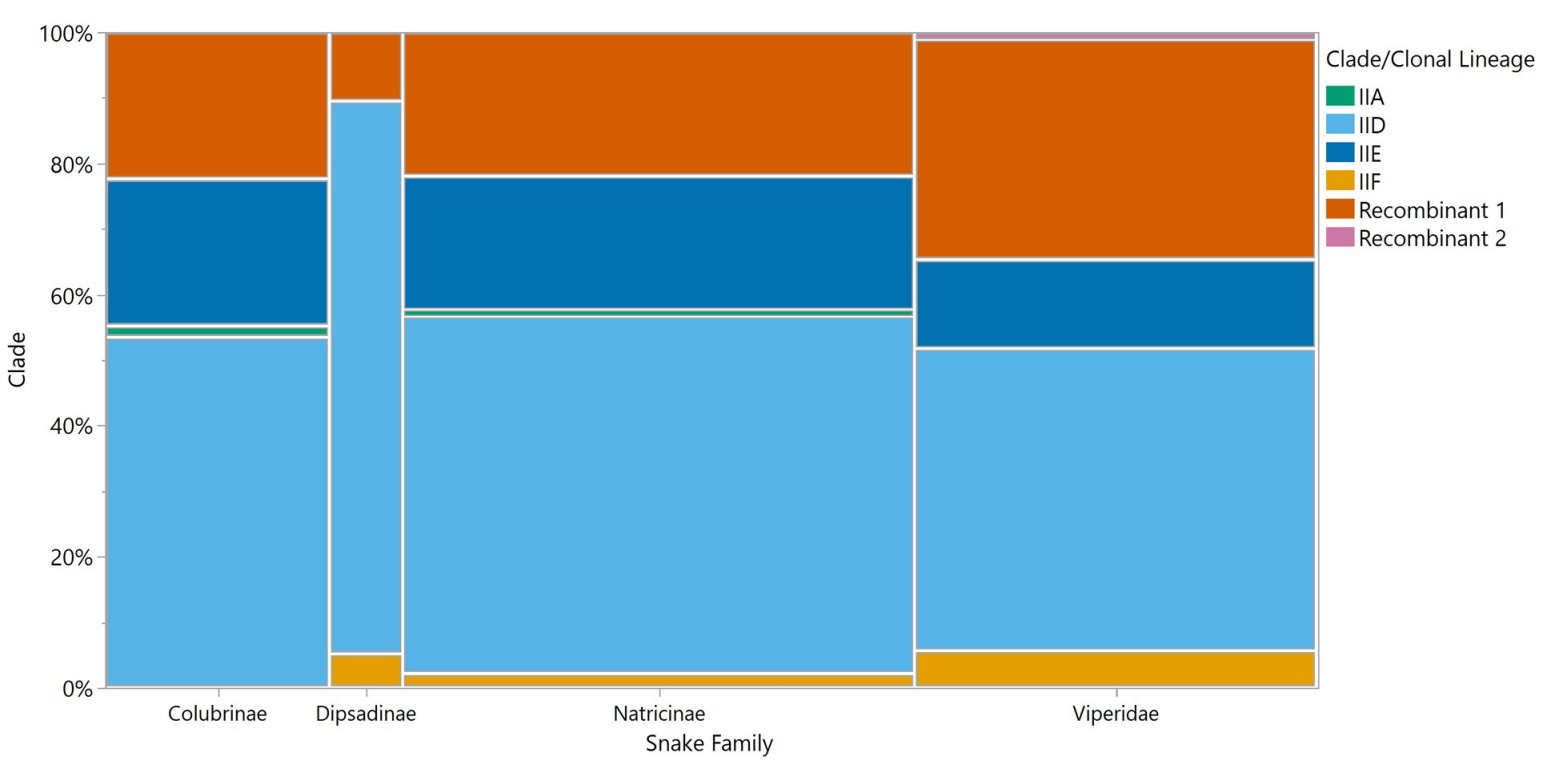
Distribution of *Ophidiomyces ophidiicola* clade/clonal lineage snake family/subfamily. Each vertical bar represents a snake family/subfamily, with the width of the bar proportional to the sample size of that group. The vertical axis shows the proportion of each clade, noted in a different color, in each group.

Most snake species were observed to harbor multiple *O*. *ophidiicola* clonal lineages, with the exception of eight species whose samples were all assigned to a single clonal lineage (Table 4 and Fig 3). However, these species were represented by six or fewer samples. When species were grouped by snake taxonomic family/subfamily, all groups had samples in clonal lineage IID and recombinant group 1, but no samples from snakes in the subfamily Colubrinae were assigned to clonal lineage IIF and no samples from snakes in the subfamily Dipsadinae were assigned to clonal lineage IIE or recombinant group 2. Only snakes from the Colubrinae and Natricinae subfamilies had samples assigned to clonal lineage IIA (Fig 4).

**Fig 5:**
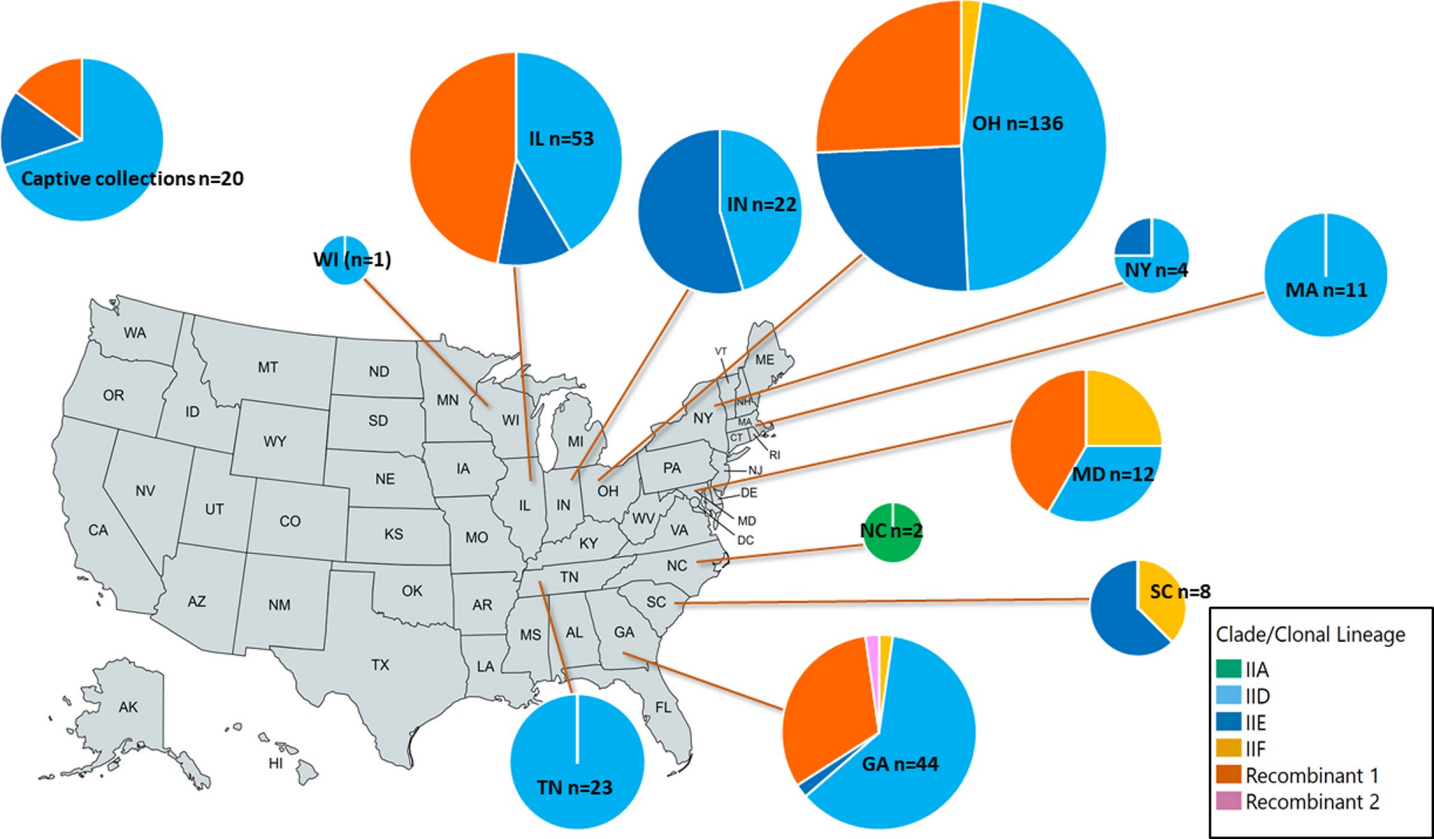
Map of the United States showing distribution of *Ophidiomyces ophidiicola* clade/clonal lineage by state. Snakes from managed collections are shown in a separate pie chart in the upper left corner. Each pie chart shows the proportion of samples from the corresponding state/collection assigned to each clade, with each clade represented by a different color. Relative size of the pie chart is representative of the sample size for the state/collection. The map outline is republished from MapChart (https://www.mapchart.net/usa.html) under a CC BY license, with permission from Minas Giannekas, founder and developer of MapChart.

Of U.S. states with more than five genotyped samples, all but two had a mix of clades, with all samples from Massachusetts and Tennessee being assigned to clonal lineage IID (Table 5 and Fig 5). Samples from snakes in managed collections were examined separately, rather than being grouped with wild snakes from the same state. Two collections had all samples assigned to clonal lineage IID, although only three samples were analyzed from each of these collections, whereas other collections had up to three different clades present, including recombinant groups. Multiple clades were detected in each year (Table 6).

Further analysis was performed for the three snake species with the highest sample sizes: timber rattlesnakes (*Crotalus horridus*, n = 31), Lake Erie watersnakes (*Nerodia sipedon insularum*, n = 86), and eastern massasauga rattlesnakes (n = 51). Genotyped samples from timber rattlesnakes from Maryland and Ohio differed in their clade assignment by state, with samples from Maryland being assigned to clonal lineage IID and recombinant group 1, and samples from Ohio being assigned to clonal lineages IID, IIE, IIF, and recombinant group 1. Samples from Lake Erie watersnakes were collected from five different nearby island sites and clonal lineages IID, IIE, and recombinant group 1 were represented on each island with varying proportions. Samples from eastern massasauga rattlesnakes were collected from two nearby, but noncontiguous, sites in Illinois and, although both sites had clonal lineage IID and recombinant group 1, only one site had samples assigned to clonal lineage IIE.

**Fig 6:**
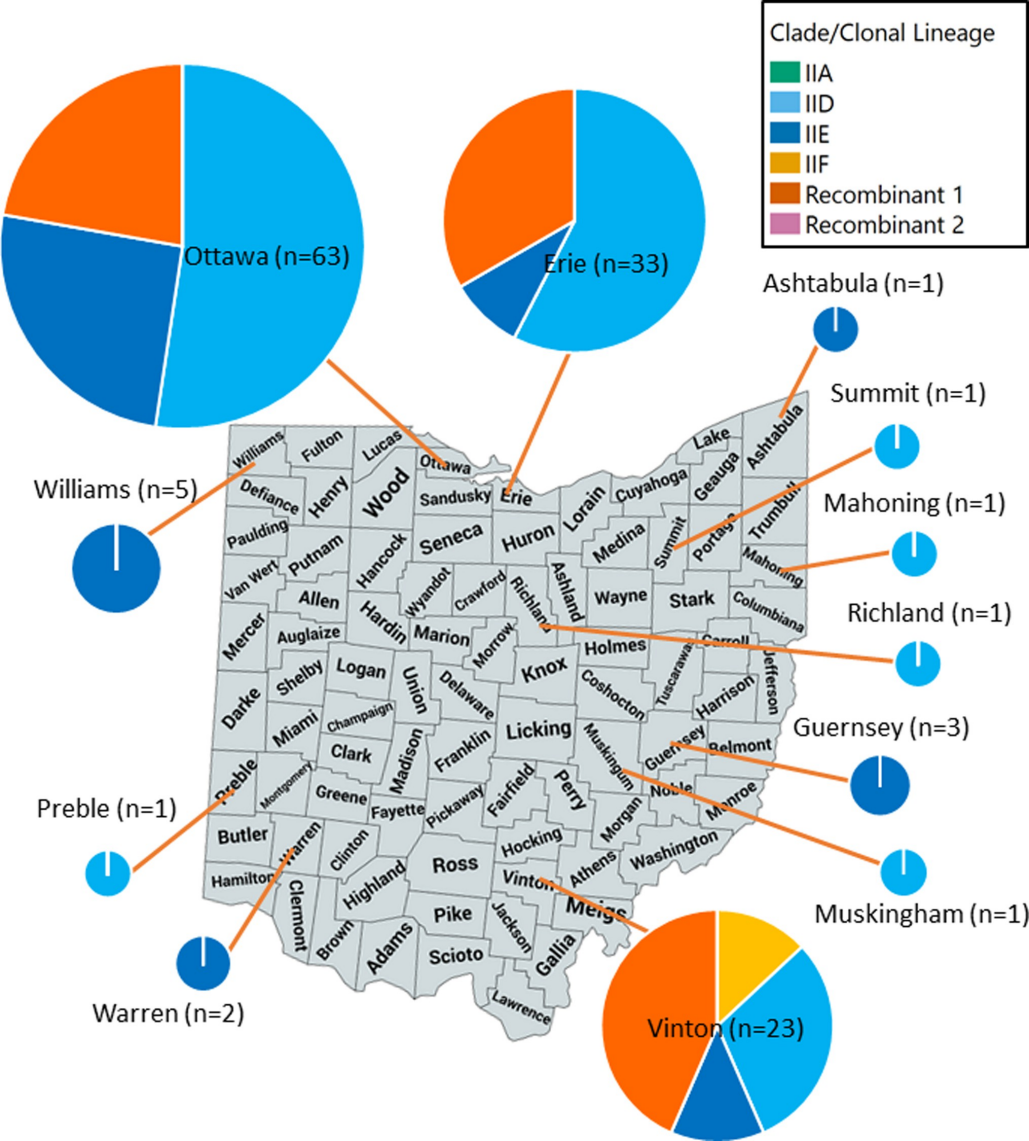
Distribution of *Ophidiomyces ophidiicola* clade/clonal lineage by county in Ohio, USA. Samples were collected between 2016 and 2020 from wild snakes (n = 137). Each pie chart shows the proportion of samples from the corresponding county assigned to each clade, with each clade represented by a different color. Relative size of the pie chart is representative of the sample size for the county. The map outline is republished from MapChart (https://www.mapchart.net/usa-counties.html) under a CC BY license, with permission from Minas Giannekas, founder and developer of MapChart.

Additional analysis was performed on results from samples from the state of Ohio, as this state had the largest sample size of wild snakes (n = 136), all of which had data for county of origin. Clade assignment varied by county, with Ottawa and Erie Counties having samples assigned to clonal lineages IID, IIE, and recombinant group 1, Vinton County having samples assigned to clonal lineages IID, IIE, IIF, and recombinant group 1, and all other counties having samples assigned to either clonal lineage IID or IIE (Fig 6).

The univariable multinomial logistic regression modeling found significant comparisons between taxonomic groups. Snakes in the genus *Crotalus* were 8.94 times more likely to have clonal lineage IIF than IID compared to *Nerodia* (95% CI: 2.03–39.36, p = 0.0045) (Table 7). Snakes in the family Viperidae were 3.98 times more likely to have clonal lineage IIF than IID compared to snakes in the family Colubridae (95% CI: 1.07–14.71, p = 0.0385) and the relative risk (RR) of assignment to recombinant group 1, compared to clonal lineage IID, was 2.08 times higher in the family Viperidae, compared to Colubridae (95% CI: 1.20–3.58, p = 0.0087) (Table 7). The relative risk of assignment to clonal lineage IIE, compared to clonal lineage IID, was significantly higher in Indiana than Georgia (RR = 32.39, 95% CI: 3.72–282.27, p = 0.0016) and the relative risk of assignment to clonal lineage IIF, compared to clonal lineage IID, was significantly higher in Maryland, compared to Ohio (RR = 16.01, 95% CI: 2.42–106.24, p = 0.0041). The relative risk of assignment to clonal lineage IIF, compared to clonal lineage IID, was significantly higher in 2017 (RR = 52.29, 95% CI: 9.47–288.65, p<0.0001), 2019 (RR = 25.75, 95% CI: 4.71–140.70, p = 0.0002), and 2020 (RR = 575.29, 95% CI: 85.89–3853.21, p<0.0001), compared to 2015 (Table 7). The best multinomial logistic regression model predicting clade assignment was moderately well supported (Akaike weight = 0.555) and included only state of origin as a predictor (Table 8). Among Lake Erie watersnakes, there were no significant associations between clade and lesion presence (p = 0.2785), number of lesions (p = 0.2997), most severe lesion type present (p = 0.1052), or disease severity (p = 0.2599).

**Table 7:** Risk ratios for significant pairwise comparisons for *Ophidiomyces ophidiicola* clonal lineages or recombinant groups by state, year, genus, and family, based on univariable multinomial logistic regression modeling.

	IIE vs IID			IIF vs IID			Recombinant Group 1 vs IID		
Level	Risk Ratio	95% Confidence Interval	p-value	Risk Ratio	95% Confidence Interval	p-value	Risk Ratio	95% Confidence Interval	p-value
Indiana vs Georgia	32.39	3.72–282.27	0.0016	NS	NS	NS	NS	NS	NS
Maryland vs Ohio	NS	NS	NS	16.01	2.42–106.24	0.0041	NS	NS	NS
2017 vs 2015	NS	NS	NS	52.29	9.47–288.65	<0.0001	NS	NS	NS
2019 vs 2015	NS	NS	NS	25.75	4.71–140.70	0.0002	NS	NS	NS
2020 vs 2015	NS	NS	NS	575.29	85.89–3853.21	<0.0001	NS	NS	NS
*Crotalus* vs *Nerodia*	NS	NS	NS	8.94	2.03–39.36	0.0045	NS	NS	NS
Viperidae vs Colubridae	NS	NS	NS	3.98	1.07–14.71	0.0385	2.08	1.20–3.58	0.0087

NS = non-significant.

**Table 8:** AICc table of generalized linear models predicting *Ophidiomyces ophidiicola* clade or clonal lineage based on state and year of sampling, as well as taxonomic family and subfamily of the snake host.

Model	K	AICc	ΔAICc	ω_i_
State	24	564.1	0.00	0.555
Family + year + state	42	565.0	0.88	0.358
Family + state	27	567.8	3.68	0.088
Genus + state	57	591.7	27.58	0.000
Genus + year + state	72	594.6	30.51	0.000
Year	18	641.4	77.33	0.000
Genus	36	642.0	77.92	0.000
Family + year	21	642.2	78.14	0.000
Genus + year	51	642.8	78.66	0.000
Family	6	645.8	81.70	0.000
Null	3	650.6	86.51	0.000

K = number of parameters, AIC_c_ = second-order Akaike information criterion, ΔAIC_c_ = difference in AIC_c_ between ranked models, ω_i_ = Akaike weight.

## Discussion

### *Ophidiomyces ophidiicola* genotyping assay performance

The qPCR-based assay designed in this study was able to assign *Ophidiomyces ophidiicola* DNA samples to eight different clades, clonal lineages, and recombinant groups based on their amplification patterns across 11 genotyping targets. The assay was highly sensitive and specific for the three major established clades, as well as three of the four clonal lineages within clade II, with a fair level of agreement with a previously described whole-genome sequencing approach [17]. The amplification patterns of clades I, II, and III and clonal lineages IIA, IID, IIE, and IIF were based on the sequences of representative genomes, whereas the patterns of recombinant groups 1 and 2 had additional target amplification, compared to the predicted patterns of the pre-established clades and clonal lineages. Although we refer to these as “recombinants,” we cannot rule out that some patterns indicative of recombinant groupings resulted from the presence of multiple clades of *O*. *ophidiicola* on a single animal, a lack of specificity of the genotyping targets, or detection of an uncharacterized strain, as noted in the parental lineages of previously described strain NWHC 44736–75 (Table 3). The explanation that they represent recombinant strains is supported by the identical amplification patterns produced by pure isolates previously established to be of recombinant origin [17]. Furthermore, recombinant groups 1 and 2 share targets with the established clonal lineages of clade II, just as recombinant strains would be expected to share certain targets with their parent strains. Recombination between strains is likely occurring within the snake populations we sampled. For example, timber rattlesnakes and Lake Erie watersnakes in Ohio had clonal lineages IID, IIE, and recombinant group 1 (Fig 3), the latter having an amplification profile indicative of both clonal lineages IID and IIE (Table 1). It is also possible that the qPCR-based assay detected the presence of multiple clades on the same animal. Mixed infections with multiple strains of the same fungi have been documented in humans, specifically with the fungi *Candida* and *Aspergillus*; however, differentiating between this and the recombinant strain hypothesis would require isolating the different strains in culture, which can be prohibitively difficult due to morphological similarities between closely related strains [48]. Although this assay was found to be highly sensitive and specific for the established clades, its ability to identify recombinant strains is limited by the unpredictable presence of the assay targets in the putative recombinant strains. Specifically, Ladner et al. [17] noted that most recombinant strains of *O*. *ophidiicola* found on snakes appeared to represent unique hybridization events and that recombination occurs throughout the genome. Thus, recombinant strains with the same parental lineages may possess different targets and produce different amplification patterns, and there are no recombination “cold spots” that can be targeted to definitively identify all parent lineages that a recombinant strain is derived from, to distinguish between the presence of multiple strains on a single animal, or to distinguish recombinant from “pure lineage” strains with a qPCR genotyping approach. In at least some instances, strains of hybrid origin could be misclassified by our assay as belonging to a non-recombinant clonal lineage. Therefore, it is important to interpret results with the knowledge that having a particular clade or clonal lineage assigned by the qPCR-based assay does not rule out that a strain could be recombinant. Conversely, the potential for cross-amplification (false positives) could result in some strains belonging to a particular clonal lineage being incorrectly designated as recombinant strains by the assay. Specifically, Target J is the only difference between clonal lineage IID and recombinant group 1, so a false positive for that target could result in misclassification. Although it is possible that some or all of the samples assigned to the Recombinant 1 group had false positive amplification of target J and are actually part of clonal lineage IID, we kept this distinction because of the possibility of recombinant strains being present and the confirmation via BLAST of the presence of target J in several of the recombinant strains assigned to Recombinant 1 group in the assay validation step (Table 3).

Of the original six strains used for assay design, three were correctly assigned (representing clades I, IIE, and III), two were incorrectly assigned (representing clades IIA and IIF), and one was not run through the assay (representing clade IID) because the DNA was not available at the time of the analysis. The two incorrectly assigned strains were further investigated using BLAST-based methods and the expected amplification patterns were produced. In each case, additional targets amplified with qPCR (targets C and K for sample 44736–89 representing clonal lineage IIA; Targets A, C, and K for sample 27466–1 representing clonal lineage IIF). Target K also had false positive qPCR amplification for two other samples, indicating that this primer-probe set may be demonstrating non-specific binding in some samples. However, qPCR amplification of Target K was confirmed by the BLAST-based approach in numerous other samples, so it is still an informative target. Finally, when the sample representative of clonal lineage IID was ‘genotyped’ using BLAST, the pattern was consistent with clonal lineage IID. Our BLAST-based genotyping results indicated that both false positive and false negative qPCR results resulted in misclassification of samples, the former being more common. False-positive qPCR amplification could have been caused by cross-contamination during sample preparation or plate loading, or by non-specific primer-probe binding. Additional comparison with WGS-based genotyping is warranted to determine if particular targets are less reliable for future genotyping applications. Samples from clades IIB and IIC were included in the agreement analysis as recombinant samples and, using BLAST-based genotyping, all had amplification patterns consistent with clade IIF. Therefore, these clades do not appear to be distinguishable from the other clades using the qPCR-based genotyping assay. Further work could be done to identify targets that would be unique to those clades and additional primer-probe sets could easily be designed and added to the assay.

Samples with lower DNA concentrations and quantities of *O*. *ophidiicola* DNA were less likely to produce amplification patterns sufficient for clade assignment. The swab-based genotyping approach for *Bd* also found that amplification was dependent on DNA quantity, with the assay performing best with a moderate amount of input DNA, approximately 150 genome equivalents/°L [28]. For our qPCR assay, which includes 2.5°L of DNA per reaction, the equivalent value would be approximately 75 ITS copies/°L. However, we measured sample DNA concentrations using a Nanodrop, which has been shown to over-estimate DNA concentrations, compared to fluorometry-based methods [49]. Therefore, our assay may be more sensitive than estimated. Another reason for lack of specific amplification could be the low quality of DNA obtained from swab samples [28], specifically that swabbing and subsequent DNA extraction can result in fragmented DNA that does not contain the full target sequences or enough target sequences for clade assignment. For some samples in our study, we found low levels of amplification of a few target sequences, but the amplification pattern was not specific for any of the clades, even with individual qPCR of sample/target combinations with Fluidigm C_t_ values between 24 and 27. Pre-amplification has been used in analyses of swab-extracted DNA to overcome the issues of low copy numbers [28] and was included in the Fluidigm protocol to increase the quantity of template DNA in this study. Although pre-amplification can increase the quantity of targets present in the sample, it cannot replace missing targets, thus the minimum genome copy requirement for this type of assay. Furthermore, pre-amplification may increase the risk of false positives by amplifying trace amounts of contaminant DNA. Some users may wish to omit the pre-amplification step depending on the amount of initial *O*. *ophidiicola* DNA in a sample or when using the assay to genotype pure cultures of the fungus.

### Epidemiology of *Ophidiomyces ophidiicola* clonal lineages

We identified the presence of multiple *O*. *ophidiicola* clonal lineages across species, states, and years, with a few exceptions, and the spatial and temporal distribution of lineages can be used to further evaluate the endemic and novel pathogen hypotheses for each of these lineages of *O*. *ophidiicola* [3, 17]. Overall, 74% of our samples from wild North American snakes were assigned to clonal lineages IID, IIE, IIF, which agrees with previous findings that these three clonal lineages represent the majority of strains detected in North American snakes and serve as the parental lineages for additional recombinant strains [17]. In this study, clonal lineages IID, IIE, and recombinant group 1 were detected as far back as 2013 and continued to be detected in 2020. Clonal lineage IID was the most geographically widespread, occurring in nine of the 11 sampled states across the Midwest, northeast, and southeast United States. Clonal lineage IIE was detected in six states in the northeast and southeast. Clonal lineage IIF was only detected in samples collected between 2017 and 2020 from four states, whereas clonal lineage IIA was only detected in two samples from North Carolina in 2018, and recombinant group 2 was detected in a single sample from Georgia in 2018. These patterns may be due to recent introduction, recent expansion of the clonal lineages, or low sample sizes for those clades. Similarly, previous work found that clonal lineage IID was the most geographically widespread, followed by IIE, with IIF having a more restricted range [17]. Although no samples in our study were assigned to clade I or clade III, the pattern of recombinant group 2 contains targets only found in clade III, indicating that it may be present in combination with other clades. Notably, our detections of clonal lineage IIA and recombinant group 2 were limited to North Carolina and Georgia, which is the region of origin for clonal lineage IIA and a strain (NWHC 44736–75) that was assigned to recombinant group 2 with our assay [17]. Clade I was defined as only including strains from wild snakes in Europe [17], so it makes sense that it was not detected among our samples from snakes in the United States. Clade III included three strains collected from snakes under human care across North America, Europe, and Australia [17] and from a wild snake in Taiwan [17, 50], so the lack of detection in our study was likely due to our limited sampling of snakes under human care. Additional genotyping of samples from the earliest detections of ophidiomycosis to present day across the United States and worldwide can provide more information about the temporal and spatial epidemiology of this disease.

The presence of a single clonal lineage in a species or population may indicate that clonal lineages differ in their ability to colonize different snake species or survive in different environments, or it may indicate recent spread of the fungus. We identified eight snake species whose samples were assigned to a single clonal lineage, but these all had small sample sizes and species was not a significant predictor of clonal lineage in the models. However, we did find that snakes in the genus *Crotalus* were at higher risk for having clonal lineage IIF detected and snakes in the family Viperidae were at higher risk for having clonal lineage IIF and recombinant group 1 detected. These taxonomic associations with clonal lineage warrant further investigation through increased surveillance and experimental infection studies. We found that eastern massasauga rattlesnakes at two sites in southern Illinois and Lake Erie watersnakes at five sites in western Lake Erie both had multiple clonal lineages present at each site, which indicates that clonal lineages overlap in their host and environmental requirements. Some collections of snakes under human care had a single clonal lineage present whereas other institutions had up to three different clonal lineages present. The presence of a single clonal lineage in a collection may indicate a single introduction, followed by spread through the collection, whereas the presence of multiple clonal lineages may indicate multiple introductions or the potential for recombination within a collection. The distribution of clades/clonal lineages was similar between snakes under human care and wild populations (Fig 2) and, for states and species with both wild and managed snakes sampled, the clades/clonal lineages detected in managed snakes tended to be present in the wild snakes as well. Analysis of these patterns is limited by the small sample size for snakes under human care in this study and would benefit from increased genotyping of *O*. *ophidiicola* samples from animals being moved between collections, which would also aid in tracking the movement of the fungus between snakes.

### Future directions

At this time, the function of the genomic areas targeted by the genotyping assay is unknown, as are differences in virulence or drug resistance between the different clades of this fungus. Differences in virulence have been associated with different strains of fungi, including *Bd* [51]. One clade of *Bd*, the global panzootic lineage, has been determined to be hypervirulent and genomic analysis found evidence of genomic recombination, potentially resulting from contact between previously isolated populations of the fungus [27]. Although no associations were found between *Ophidiomyces* clade and disease severity metrics in Lake Erie watersnakes, clinical signs of ophidiomycosis have been found to vary over time [52–54] and the snakes included in this analysis may have been at different points in their disease progression at the time of observation, which makes it difficult to compare the severity of clinical disease caused by each fungal clonal lineage. Furthermore, because the assay may not detect all instances of recombination, it is possible that genotyping results do not capture the presence or absence of specific genes that may be involved in greater virulence. Increased use of standardized, quantitative methods for evaluating disease severity in snake populations, along with experimental infection studies to assess differences in strain infectivity and pathogenicity, would enable better identification of genetic markers associated with virulence or drug resistance. If such markers exist, our method could be further adapted to target those areas, such that genotyping at the first detection of infection could help to predict the severity of disease, facilitate selection of appropriate treatment, predict the length of treatment needed, and indicate the prognosis for recovery, with or without treatment.

Overall, this qPCR-based genotyping assay represents a new technique to explore the epidemiology of ophidiomycosis using *O*. *ophidiicola* DNA extracted from skin swabs. Assigning samples to clades based on their amplification patterns decreases the time and effort necessary to analyze results, compared to techniques that involve sequencing, and the use of the Fluidigm platform allows for the processing of large numbers of DNA samples in a short time using minimal volumes of DNA and primer-probe. Although the method may not accurately assign all strains to a particular clade or lineage, the assay has value in investigating strain diversity within snake populations or geographic regions, investigating correlations between particular genotypes and disease outbreaks, and identifying possible relationships between genotypes and pathogen characteristics. Additionally, the design of this assay allows researchers to add and/or remove DNA targets as our knowledge of *O*. *ophidiicola* phylogeny and ophidiomycosis etiology evolves. Finally, by furthering our understanding of the roles various clades/clonal lineages of *O*. *ophidiicola* play in ophidiomycosis epidemiology, application of this assay will allow for development of management practices to mitigate the effects of this disease on snake health.

## Supporting information

S1 Table Results of detection sensitivity testing for each of the 11 primer-probe sets selected for the genotyping assay, including the limit of detection, slope and coefficient of determination (R^2^) of the best-fit line, and reaction efficiency. (PDF)

S2 Table Target sequences used to design each of the 11 primer-probe sets used in the qPCR-based genotyping assay for *Ophidiomyces ophidiicola* and for BLAST-based genotyping of whole genome assemblies of selected samples used in assay validation. (PDF)
