# Supplementary material for: Development and application of a qPCR-based genotyping assay for Ophidiomyces ophidiicola to investigate the epidemiology of ophidiomycosis
Source: PLoS One. 2023 Aug 3;18(8):e0289159. doi: 10.1371/journal.pone.0289159 (PMC10399865; doi:10.1371/journal.pone.0289159)
Supplement: S1 Table — (PDF) [file pone.0289159.s001.pdf]

S1 Table. Results of detection sensitivity testing for each of the 11 primer-probe sets selected for the genotyping assay, including the limit of detection, slope and coefficient of determination (R^2) of the best-fit line, and reaction efficiency.

Target ID	Limit of detection (target copies per µL)	Slope	R^2	Reaction efficiency
**A**	1	-3.204	0.994	105.168
**B**	1	-3.227	0.992	104.115
**C**	10	-3.454	0.998	94.788
**D**	10	-3.482	0.998	93.716
**E**	10	-3.588	0.999	89.986
**F**	10	-3.541	0.999	91.608
**G**	1	-3.263	0.990	102.502
**H**	10	-3.306	0.998	100.673
**I**	1	-3.340	0.993	99.235
**J**	10	-3.387	0.999	97.354
**K**	10	-3.422	0.996	95.994
