# Supplementary material for: Development and application of a qPCR-based genotyping assay for Ophidiomyces ophidiicola to investigate the epidemiology of ophidiomycosis
Source: PLoS One. 2023 Aug 3;18(8):e0289159. doi: 10.1371/journal.pone.0289159 (PMC10399865; doi:10.1371/journal.pone.0289159)
Supplement: S2 Table — (PDF) [file pone.0289159.s002.pdf]

S2 Table. Target sequences used to design each of the 11 primer-probe sets used in the qPCR-based genotyping assay for *Ophidiomyces ophidiicola* and for BLAST-based genotyping of whole genome assemblies of selected samples used in assay validation.

Target ID	Sequence
**A**	ACCTGCAGATGATATGGAGCAGGCGCACACTTTCCAAAAAACACAGTTTGAATTTTCTTTC<br>ACTGCATCTAATAAACGATCGTGTTTACAGCCCCATTGCGCAAGTAACGAGACAAGAGAT<br>ACTCTGTTTGCTTCTATAAATGGCAACGGGGGTGAGCACTCCTCTAGATCAAGCATAACAG<br>AACCCCCTCCACCTGTACACTCACCCGAAGCCGAAGAAACACCAGCCGTCTTGAATAAC
**B**	CTGCCGTAGTTACATAGTTACATATTGGCGCCGTCCGGAATGCGTTTTGGCCATTCAACTC<br>TAACCCGACAAACCACAAATATGGATAACTACTACTGTACCTTGTACTCCCTGTACGCGCC<br>GGCTGTGTGCGGTTATAGTTCACCAGCCTGTATAAAGAGGGCCAGCTCCCTCAAGTTCTCT<br>CCTTATCTTGCATGTTGTGACAATCTCCCCTAGAGCCCTTTACACCATTCCGTTTTTTTATC<br>CTCTTTATTTTGCTCAAATGCGTGGTCCAAAGGTCTACGATAACTGGATCGGTAACATGTG<br>GAACAATTACAAGCTCCGCTGCCCTGATGGAACA
**C**	ACACGTTGTAAAGTAGTCACGCACTGCCATTCGAAATAGTGGCTGATATCCATATCTTTTC<br>ACATAGTGTTGTAGCATCTCATTTGCTCGGTCCTGCTTGACTGCTAGTTGCACCCTAAACA<br>GTTCCAAAAGCTCGGGATTTTCATCTTGAATTTCAGAAAGATCAAGAAGATGTGTTAGATC<br>AATCTTTCCACCCATAAAATCTTCTGCATC
**D**	TCATTTTCACACAGGCTCTCCTCACACAATCCAATACTCATAAATCTGAAGCAAATAAACT<br>TTTCTTATCCGCGTCTTACTCGGAGGCTATATCAACCTACGATCGTGCCTTAGCGTTTTGCC<br>CCAGCTATCTCGACTACGAAATCGCAGTACTGCGGAGCAACATTGCCGCATGCCACTTGA<br>AACTTGAAGATTGGAAGCTTGCCATAGATGCTGCAACTGCCAGCATTGCATGTTTGGATCG<br>AGTGCTTCCTTCCCTTTCTCCTGTTACCTCTGCGTTGGAACAGGGAAACGGTTTTTGCG
**E**	GGCCCGATGGAATCAGAAAGACGATGACCTGCTGGAGCGCCTCCGGGCCCGCGGCTGGAG<br>TTGGTGGGAGATCAAACAACAGTTCCCAGGCCGAACGTTACCCGCTCTGCAGCAGCGACA<br>CATGAAGCTTCAGGCGATGGGAAATGGTCAGTGCCCCAAAGATGAGTGATAGCGCGCATG<br>CCATTATGGAGCTGGAGTCTGCAAGCCAAGAGCACGACCACAGGGAGGCAGCAATGCGTC<br>AGCGGTATTTCAACCAGAGAATCCCAAGGAACTTTCACCGTTAACCAAGGGCGTGATCTT<br>ACACCAAGTGCTG
**F**	TACTCCGCTGCCCGCCATTCCGGAGATTTCAACCCTTGACCATCAAATCTTCCTGAATGGA<br>GGGACTACCTCTAAACTCGAACGGCTACCATCACGGCTGCAGTCTCATAAAAAGAGATCC<br>TCATCCTTGTACTCTACCGATCAGCTCCACGATCTGTCGTATGAGAATTCATACCTCAGGG<br>GAGAACTGTCGCGGCTCGAGGAGTACCGATATGCACTTTTGAA
**G**	ACAGGCGAGCCCTTGCCCTTTTGCTTTCCATTAAAATCAGGTTGCGATCCAAAATCAGATA<br>CCACTATAGGTTGAGCAGCTCTCGTCTTAAAAAATCAGATGAAATGGGTAACAAAGTTTG<br>CTCGCAATCCCGACCATCCTGCGCAACTACAAAAGACCTTGTTGTTAGCCATGGTTGCAAA<br>CACTGACAGCCACTCTAGAATTGTCCTTACCTGAAAATGAGTCAAGATCACAGCAGAGAC<br>AATAGAAGCAATCAGGACTATCCCTGCGAAGATATTACCCGGCCTCAGTTTACTCCGCTGC<br>CCGCCATTCCGGAGATTT
**H**	GTGACCATTGTGGGCTTTTATACTCATTTGTAGTTGTGAACACTCAATGAAGATGAGTTTT<br>ATCATGCAAGCTTATTCTTTCGCAAGAGAATAATACAGTACTAGGTCAAAAGTGTTGACAC<br>CCCCCCTACTTTTACACCAAAACCCACTCTTTTCACAACCAAGTTGCTCAACAAAATTGTA<br>TTGTAACATATCAATTAATACACATTAATATGTAGAGAATTGGTAGAAGAATACTTTAAGA

ATAATTTTATTAAATAATTATAAATTATTGTCTCTGTAAATCTCATTAAATATGACTGAAA
GCTATCATCACATAGACTCCTCTATACATCATGAAAATAAGTCAT

I GGACTTTGATGGTAGCGATAATAGCATGAGTACCCCCATTCTTCTCCCTCCAAACCTTCCA
TGATTTGGGAATTTTGTTGCCTGCGTTAACTTGAACCTGAAAACGGTGGAACTTCTTGCCA
GCCTCCTCCTCGGTCCCAGCGAAATCGAAACGAACGCCCAACTTTTCCTGGTCCTGTGCGG
TTTTCGGATAGGCGGTATGATGAAGGCTGACGGTTGGGCGGAGGAGCTTTTTGTAGCCGC
CTTTCATAACAAAATCTTTGACTTTGTTCCACTTGTTAGGCATGGTGAAGGTGTTCTAATCT
CTAAGTTTAAATCGAG

J CACTAAATAGGGCAATAGAGACAAGTTTCATTATGTCTTCATATTCCTAGAGAGCTATCAA
CACTATGCGTCCAACTTAAAAATAGAGAAAAAAATCTCAAGGTCACTAATATAAGATAGAA
AGAAATTTGACCCCCAGAAACACTTACGCACAACGCACTGCCGTTTAGGTCTCTTCCCGCG
ACCTAAGCCCTGAATCTCAGCCTTCCGCTTCCGAGTGCGTGAAAGAGGCGGACGTGAGTC
GCAGCCGGAGCAGTGTAGCTGGCCAGGGGTACCATGGTCTGGCTGCCCAAAACACATGTC
AAATCCTTGCTGCTCCACGAATCGA

K TTACTATAGGCAAAAACTAAATCTAGTATAAAATTAAAGGCTTGTAGATATTATAATAGTA
GTTATTAACTAACTAGTTACTGCAGTAACTACCAAGATATAAAGAGCGAGACATGTCAAG
AGTCGTGGGAAAAGTGAACGTAGAAGCTCATGGAACTGTATCGAAGGTACTTCGCTCGCT
GGTCTAGAAACGTCACATAAGATGCTCGTCCCTCTTCGAGCCCTTCTCCATGTTCCTTCATC
AGGCTACCACGCTTCCTCACCATGCTCGAGTCCTCGCCCGCAGTATTTCGGGCTTATCCTC
CAAGGTTGCGGATGTGAGACGATGAGAGCAACCACAAAGGCATATATAGGTTAATTCACG
ATATGAATAACACTAGCTCAAACGCA
